# Comprehensive Responses of Physiology and Rhizosphere Microbiome to Saline–Alkaline Stress in Soybean Seedlings with Different Tolerances

**DOI:** 10.3390/plants14223480

**Published:** 2025-11-14

**Authors:** Bikun Wang, Fangang Meng, Tong Cheng, Jiarui Niu, Demin Rao, Zhe Han, Wei Zhang, Zhian Zhang

**Affiliations:** 1 College of Agronomy, Jilin Agricultural University, Nanguan District, No. 2888 Xincheng Ave, Changchun 130118, China; wangbikun@outlook.com (B.W.); 19334492365@mails.jlau.edu.cn (J.N.); 2 Soybean Institute, Jilin Academy of Agricultural Sciences, Nanguan District, No. 1363 Caiyu Ave, Changchun 130033, Chinachengtong@jaas.com.cn (T.C.); raodemin@jaas.com.cn (D.R.); 3 College of Agronomy, Yanbian University, No. 977 Gongyuan Rd., Yanbian Korean Autonomous Prefecture, Yanji 133002, China

**Keywords:** soybean, salinity, abiotic stress, salinity tolerance, rhizosphere microbiome, metagenomics

## Abstract

Soil salinization severely threatens global crop production. Understanding the relationship between crop saline–alkaline tolerance physiology and the rhizosphere microbiome, and leveraging beneficial microorganisms to enhance crop stress resistance, holds importance for sustainable agricultural development. This study investigated the physiological and rhizosphere microbial responses of two soybean cultivars with different saline–alkaline tolerance to stress. Under saline–alkaline conditions, the tolerant cultivar exhibited superior physiological performance, including higher chlorophyll content, photosynthetic efficiency, and elevated activities of antioxidant enzymes (SOD, POD, and CAT), alongside reduced oxidative damage (MDA) and greater biomass accumulation. Combined metagenomic and physiological analyses revealed significant correlations of *Bradyrhizobium* and *Solirubrobacter* with key physiological indicators, including dry weight, PI_ABS_, φ_po_, and MDA. The tolerant cultivar selectively enriched distinct marker microbes, such as *Bradyrhizobium* sp. and *Bradyrhizobium liaoningense*, in its rhizosphere. We conclude that the tolerant cultivar exhibits strong intrinsic physiological resistance. This resistance is further enhanced by a beneficially assembled rhizosphere microbiome, while the host plant’s physiology remains the dominant factor.

## 1. Introduction

Soil salinization poses a major challenge to global agricultural development. Human activities and global climate change are exacerbating soil salinization. It is estimated that the global area of saline–alkali land is approximately 1 billion hectares and shows an upward trend, severely hindering agricultural sustainability and threatening food security worldwide [1,2]. The accumulation of soluble salts in the topsoil leads to adverse changes in soil physicochemical properties and structure, manifested as poor water and air permeability, decreased soil enzyme activity and fertility, and highly detrimental effects on crop growth. Soybean is a globally important oilseed crop, providing 30% of the oil in the human diet, with global annual consumption exceeding 300 million tons [3]. Against this background, to improve and utilize saline–alkali soils, studying soybean adaptation strategies under saline–alkali stress and further enhancing their saline–alkali resistance is of great significance.

Photosynthesis is the fundamental process for organic matter synthesis in crops, and saline–alkali stress significantly affects the photosynthetic performance of functional leaves, thereby influencing crop growth and yield formation [4]. Previous studies have shown that parameters derived from chlorophyll fluorescence kinetics (OJIP) curves and chlorophyll content measurements can be used to assess the physiological health of crop leaves. In particular, chlorophyll fluorescence is highly sensitive to various abiotic stresses, including saline–alkali stress [5]. In addition, saline–alkali stress exerts osmotic and ionic toxic effects on plant roots, disturbing the dynamic balance of reactive oxygen species (ROS), increasing biomembrane permeability, and ultimately suppressing plant cell physiological functions [6]. Malondialdehyde (MDA), a product of membrane lipid peroxidation, serves as a marker for the extent of biomembrane damage [7]. In response to intracellular ROS accumulation, plants activate antioxidant enzyme systems, including SOD, POD, and CAT, to scavenge ROS, maintain cellular redox homeostasis, and mitigate cell damage to sustain normal physiological functions [8,9,10]. Measuring the activities of different antioxidant enzymes provides an effective way to evaluate plant stress resistance. Previous research has yielded abundant findings on plant physiology under saline–alkali stress. Concurrently, with the increasing development of high-throughput sequencing technologies in recent years, the role and contribution of the plant rhizosphere microbiome have been gradually revealed. The rhizosphere microbiome is now considered a key component of plant responses to abiotic stress, often regarded as the plant’s “second genome.” Rhizosphere microorganisms and plant roots interact closely; plant roots provide nutrient sources for microorganisms through root exudates and can selectively recruit beneficial microorganisms by modulating the composition of these exudates to promote plant growth and development [11]. In return, microorganisms enhance plant nutrient acquisition (e.g., rhizobia directly provide nutrients to hosts through nitrogen fixation). Under stress conditions, plants can recruit beneficial microorganisms and alter the local root microenvironment to enhance their stress tolerance [12]. Previous studies have confirmed that bacteria in the rhizosphere microbiome play a crucial role; bacteria beneficial for plant growth while enhancing plant stress resistance are termed Plant Growth Promoting Rhizosphere Bacteria (PGPR) [13]. The recruitment of the soil rhizosphere microbiome is influenced by the plant’s genetic background, leading to distinct microbial community differences among genotypes. These variety-driven differences in microbial communities are important factors affecting their stress resistance [14]. Soil type and host genotype jointly determine the rhizosphere microbial community and function. Genetic differences between tolerant and sensitive varieties lead to differences in their root exudates, subsequently recruiting different microbial communities and forming differences in stress resistance [15]. Identifying microorganisms that can enhance plant resistance and their recruitment pathways is crucial for understanding the intrinsic mechanisms of plant sensitivity to stress.

The great potential of microbiomes in enhancing crop stress resistance suggests that deliberately shaping the crop rhizosphere microbiome could become a promising strategy to rapidly improve stress tolerance. Currently, research on how the rhizosphere microbiome and soybean physiology synergistically respond to saline–alkali stress remains limited. The seedling stage is a critical period for soybean growth under saline–alkali stress. Therefore, we selected two soybean varieties, Jiyu 99 (tolerant) and Jiyu 83 (sensitive), which exhibit significantly different levels of saline–alkali tolerance, to investigate how genotypes with contrasting tolerances coordinately respond to saline–alkali stress during the seedling stage. The focus of this study was as follows: (1) examine the physiological responses of seedlings to stress in soybean varieties with different levels of saline–alkali tolerance; (2) determine whether these varieties shape distinct rhizosphere microbial communities under salt stress; and (3) identify dominant microbial taxa specific to the tolerant variety and explore the associations between seedling physiology and the rhizosphere microbiome across genotypes with differing tolerances. This study further explores which factor contributes more critically to this synergistic interaction system—the soybean’s intrinsic stress-resistant physiology or the rhizosphere microbiome.

## 2. Results

### 2.1. Differences in Overall Growth Status and Functional Leaf SPAD Values

**Figure 1:**
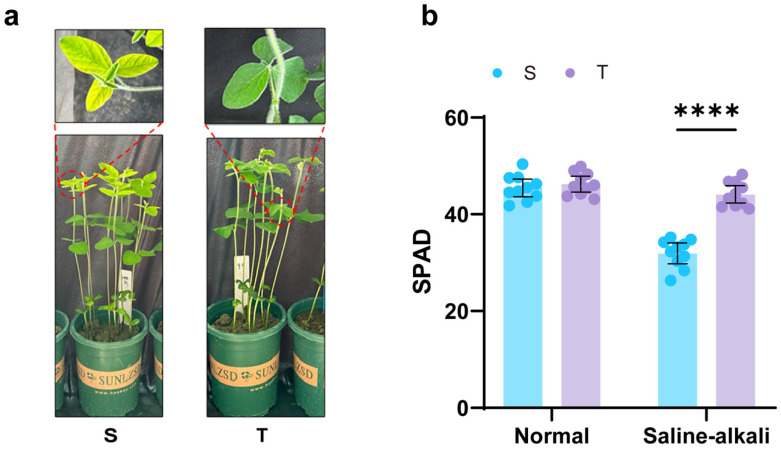
Growth status and functional leaf SPAD values of different varieties under saline–alkali stress. (**a**) Growth performance of different varieties under saline–alkali soil conditions. (**b**) Differences in functional leaf SPAD values between varieties. Note: T is the tolerant variety, S is the sensitive variety, the dots above the bars represent the values of individual samples measured for SPAD, n = 10, **** represents *p* < 0.0001.

The salt-tolerant variety (T) exhibited better growth status in saline–alkali soil compared to the salt-sensitive variety (S), with the latter showing obvious chlorosis in functional leaves and unexpanded new leaves (Figure 1a). Comparative analysis of functional leaf SPAD values between varieties under the two soil conditions showed no significant difference under normal soil conditions. However, under saline–alkali stress, the SPAD value of T was significantly higher than that of S, by 38.0%. Compared to normal soil, SPAD values in both varieties showed a decreasing trend in saline–alkali soil, with the decrease being greater in S (29.7%) than in T (4.5%) (Figure 1b). These results indicate that saline–alkali stress adversely affects the chlorophyll content of soybean functional leaves, with a more severe impact on the sensitive variety.

### 2.2. OJIP Fluorescence Rise Kinetics Curve and JIP-Test Results

**Figure 2:**
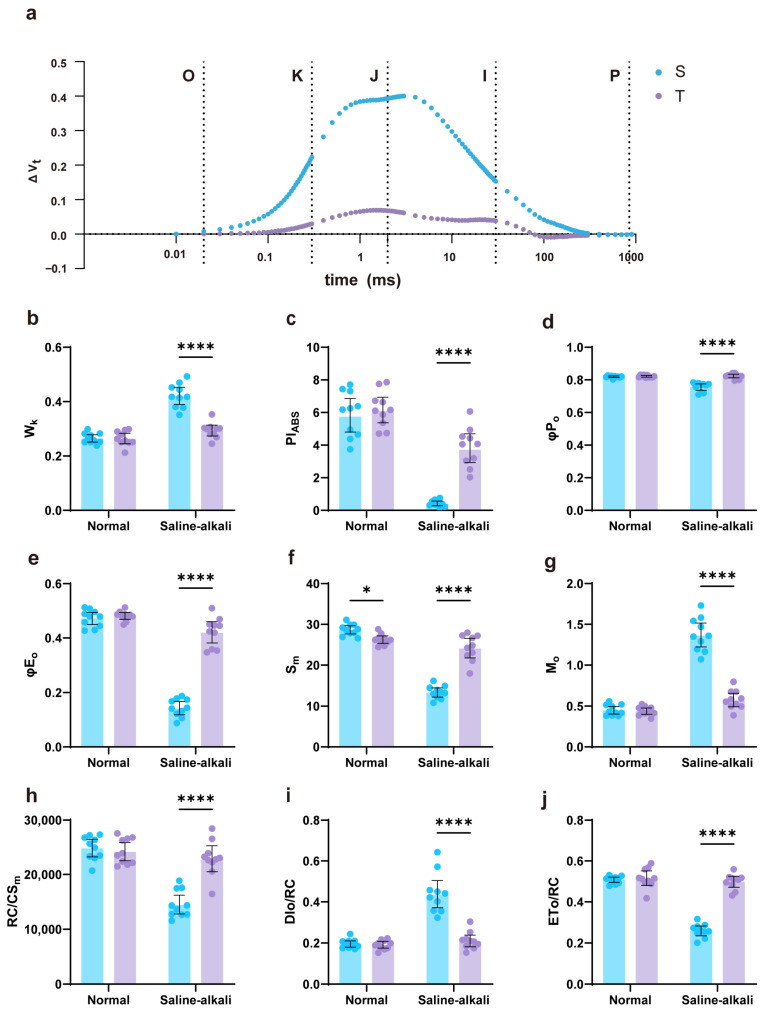
Chlorophyll fluorescence kinetics curves and selected parameters. (**a**) O-P normalized OJIP transients showing standardized variable fluorescence (Vt), where Vt = (Ft − Fo)/(Fm − Fo) and ΔVt = VtS − VtN (VtS is Vt under saline–alkali stress, VtN is Vt in normal soil). Labels indicate O-, K-, J-, I-, and P-phases. (**b**) W_k_ = (F_K_ − Fo)/(F_J_ − Fo). (**c**) Photosynthetic performance index: PI_ABS_ = [γRC/(1 − γRC)] × [φP_o_/(1 − φP_o_)] × [ψo/(1 − ψo)]. (**d**) Maximum quantum yield of primary photochemistry φP_o_ (equivalent to Fv/Fm). (**e**) φE_o_, probability that an absorbed photon moves an electron into the electron transport chain beyond QA^−^. (**f**) S_m_, area under the OJIP curve. (**g**) M_o_, initial slope of fluorescence. (**h**) RC/CS_m_, number of active reaction centers per unit area. (**i**) DIo/RC, energy dissipated per reaction center. (**j**) ETo/RC, electron transport flux per reaction center. Note: T is the tolerant variety, S is the sensitive variety, the dots above the bars represent the values of individual samples measured for each parameter, n = 10, where * represents *p* < 0.05, **** represents *p* < 0.0001.

To investigate the differences in photosynthetic efficiency of functional leaves between the two soybean varieties under saline–alkali stress, we measured the OJIP fast fluorescence rise kinetics curve and performed JIP-test analysis. The results of the O-P normalized OJIP curves are shown in Figure 2a. Under saline–alkali stress, both varieties showed a distinct K-band, and the fluorescence intensities at the J and I phases increased significantly, indicating that stress affected the function of photosystem II (PSII). The salt-sensitive variety (S) had higher fluorescence intensities at the K, J, and I phases than the tolerant variety (T), indicating more severe PSII damage. The W_k_ parameter reflects the damage degree of the PSII donor-side oxygen-evolving complex (OEC). As shown in Figure 2b, under normal soil conditions, there was no significant difference in W_k_ values between S and T. Under saline–alkali stress conditions, the W_k_ value of S was significantly higher than that of T, by 43.3%. Compared to normal soil, the W_k_ values of both T and S increased under saline–alkali stress, with T increasing by 11.2% and S by 59.1%, indicating that saline–alkali stress caused OEC damage, with S being more severely affected. PI_ABS_ is a comprehensive index characterizing the overall performance of the photosynthetic apparatus. As shown in Figure 2c, there was no significant difference in PI_ABS_ between the two under normal soil conditions. Under saline–alkali stress, the PI_ABS_ value of T was significantly higher than that of S, differing by as much as 777.6%. Compared to normal soil, the PI_ABS_ values of both T and S showed a decreasing trend, with T decreasing by 37.1% and S by 92.5%, indicating that saline–alkali stress significantly inhibited photosynthetic function, with S being more severely affected. φP_o_ represents the efficiency of PSII reaction centers capturing light energy, equivalent to the maximum photochemical efficiency (Fv/Fm). Basic parameters such as φE_o_ (efficiency of electron transport beyond QA^−^), the initial slope of fluorescence M_o_, and the area under the OJIP curve S_m_ showed trends consistent with parameters like W_k_ and PI_ABS_ (Figure 2c–g): T and S showed no difference or small differences under normal soil conditions, while differences appeared or increased between T and S under saline–alkali stress. Parameters such as the number of active reaction centers per unit area (RC/CS_m_) and energy flux parameters (DIo/RC, ETo/RC) also showed similar patterns (Figure 2h–j). Combined, these results indicate that saline–alkali stress leads to a decline in the overall performance of the photosynthetic apparatus in soybean leaves, specifically manifested as OEC damage, reduced reaction center activity, weakened electron transport capacity, and decreased light energy utilization efficiency of reaction centers. Compared to the tolerant variety T, the PSII function of the sensitive variety S was more adversely affected.

### 2.3. Differences in Root Oxidative Stress Response and Antioxidant Enzyme Activity

**Figure 3:**
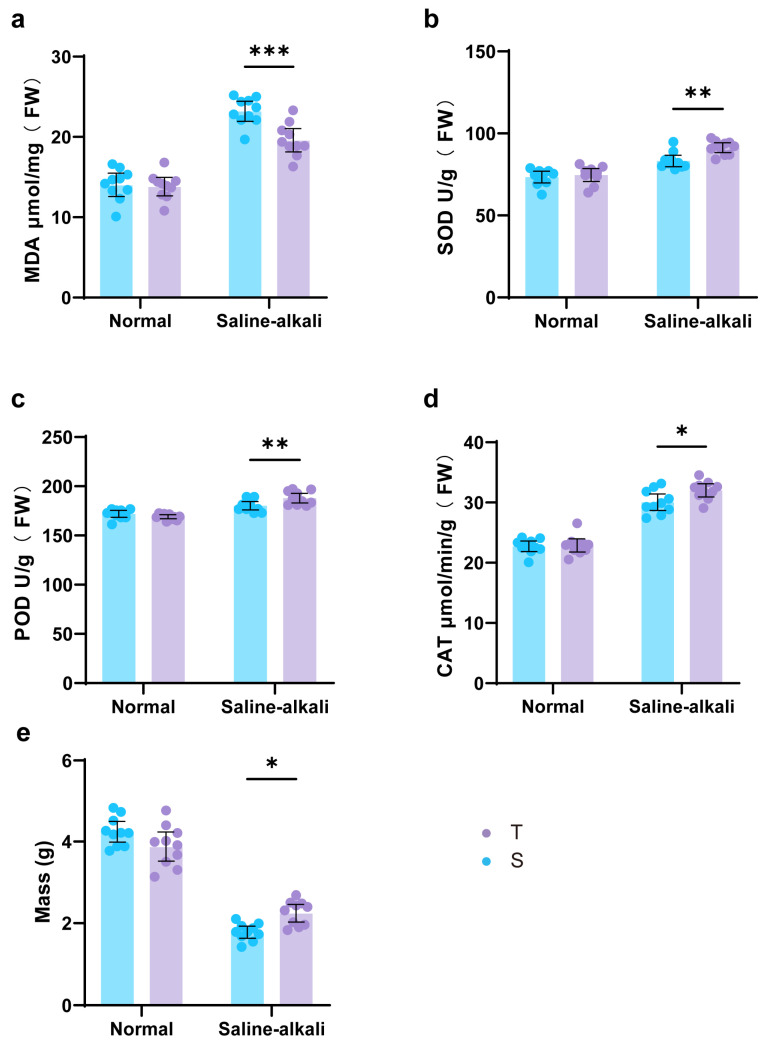
Root MDA content, antioxidant enzyme activities, and whole plant dry matter accumulation. (**a**) Malondialdehyde (MDA) content. (**b**) Superoxide dismutase (SOD) activity. (**c**) Peroxidase (POD) activity. (**d**) Catalase (CAT) activity. (**e**) Whole plant dry matter accumulation. Note: T is the tolerant variety, S is the sensitive variety, the dots above the bars represent the values of individual samples measured for each parameter, n = 10, * represents *p* < 0.05, ** represents *p* < 0.01, *** represents *p* < 0.001.

Malondialdehyde (MDA) is a key indicator reflecting the degree of membrane lipid peroxidation and can be used to assess the stress level in plant tissues. Under normal soil conditions, there was no significant difference in root MDA content between the two varieties (Figure 3a). Under saline–alkali stress, the MDA content in the roots of S was significantly higher than that of T, by 18.2%. Compared to normal soil, the root MDA content of both varieties increased (T: 41.7%; S: 64.5%). The above results indicate that saline–alkali stress intensified membrane lipid peroxidation in soybean roots, leading to a significant increase in MDA accumulation, with the salt-sensitive variety (S) being more severely affected.

Under saline–alkali stress, the activities of SOD, POD, and CAT in both S and T varieties increased significantly (Figure 3b–d), indicating that stress conditions activated the root antioxidant enzyme system. Further comparison revealed that the activities of SOD, POD, and CAT in the tolerant variety T were significantly higher than those in S by 9.6%, 4.3%, and 6.5%, respectively (Figure 3b–e). This suggests that T more effectively scavenged reactive oxygen species (ROS) by specifically enhancing the above antioxidant enzyme activities, thereby mitigating oxidative damage, which is consistent with its lower MDA accumulation level.

In summary, saline–alkali stress induced membrane lipid peroxidation in soybean roots and activated antioxidant enzyme systems such as SOD, POD, and CAT. The tolerant variety T effectively reduced the degree of membrane lipid peroxidation by maintaining higher antioxidant enzyme activities, resulting in lower accumulated MDA content, thus exhibiting stronger saline–alkali resistance.

### 2.4. Differences in Dry Matter Accumulation

As shown in Figure 3e, compared to normal soil conditions, the dry matter accumulation of both soybean varieties decreased significantly under saline–alkali stress, with the salt-sensitive variety (S) decreasing by 58.0% and the tolerant variety (T) by 42.1%. Furthermore, under saline–alkali stress, the dry matter accumulation of T was significantly higher than that of S, by 26.1%, indicating that the tolerant variety maintains a stronger dry matter accumulation capacity under stress conditions.

### 2.5. Analysis of Rhizosphere Microbial Community Structure and Correlation with Physiology

**Figure 4:**
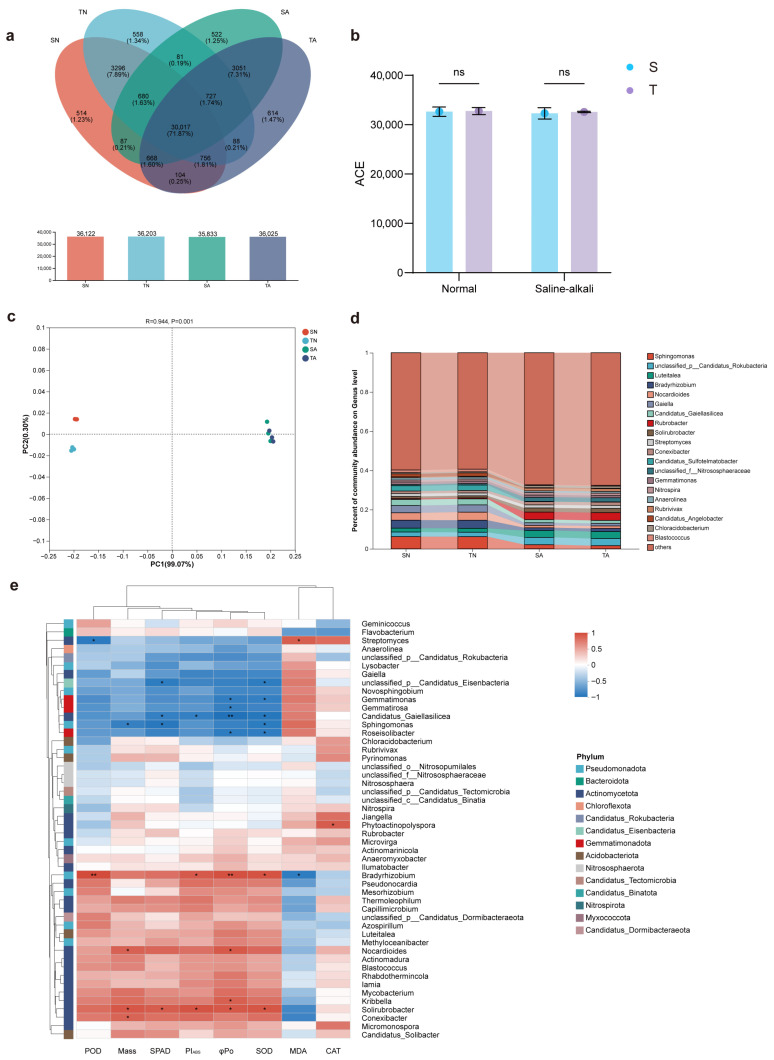
Microbial community structure and diversity under different soil conditions. (**a**) Microbial abundance composition in different rhizosphere soils (species level). (**b**) Alpha diversity based on the ACE index. (**c**) Beta diversity analysis: PCoA based on Bray–Curtis distance, inter-group differences tested using ANOSIM. (**d**) Changes in species abundance at the genus level. (**e**) Correlation between the top 50 abundant genera in saline–alkali rhizosphere soil and physiological indicators and dry matter, * represents *p* < 0.05, ** represents *p* < 0.01.

To study the association between soybean physiology and its rhizosphere microbial community under saline–alkali stress and to analyze the role of rhizosphere microorganisms in combating stress, we performed metagenomic sequencing analysis on the rhizosphere microbial communities of two soybean varieties with different saline–alkali tolerances. According to the annotation results, at the species level, 36,122, 36,203, 35,833, and 36,025 species were annotated in the SN, TN, SA, and TA groups, respectively. Among them, 30,017 species were common to all four groups, accounting for 71.9% of the total (Figure 4a), indicating that the core microbiome at the species level was highly similar across different soil treatments. Alpha diversity analysis based on the ACE index showed no significant differences in alpha diversity between the same variety in different soils or between different varieties in the same soil, indicating that rhizosphere microbial richness was comparable for the same variety across different soils and for different varieties in the same soil (Figure 4b). Beta diversity analysis based on Bray–Curtis distance PCoA showed differences in microbial structure between groups, where PC1 explained 98.33% of the variance and PC2 explained 0.40% of the variance. ANOSIM analysis results showed extremely significant differences in microbial communities between groups (R = 0.9599, *p* < 0.001) (Figure 4c). Rhizosphere soils of the same soil type were closer on PC1, while rhizosphere soils of the same variety showed greater similarity on PC2. This indicates that the difference in rhizosphere soil microorganisms mainly comes from soil type, and the difference in rhizosphere microorganisms between different varieties under saline–alkali soil is smaller than that under conventional soil, i.e., under saline–alkali stress, the rhizosphere microorganisms of the two varieties tend to converge overall.

To clarify the overall impact of saline–alkali stress on the soybean rhizosphere microbial community, we analyzed the species abundance of rhizosphere microorganisms at the genus level. At the genus level, microbial abundance showed obvious differences with soil type. Under the same soil conditions, microbial abundance at the genus level was similar between different varieties. In saline–alkali rhizosphere soil (TA, SA), stress-tolerant actinobacterial genera such as *Rubrobacter* and *Solirubrobacter* were significantly enriched. In contrast, conventional rhizosphere soil (TN, SN) enriched genera related to organic matter decomposition and nitrogen fixation, such as *Sphingomonas*, *Bradyrhizobium*, and *Gaiella* (Figure 4d). The differences in rhizosphere microbial communities between different soil types indicate that saline–alkali stress may inhibit microbial taxa involved in important functional processes such as organic matter degradation and nitrogen fixation, while promoting those with saline–alkali tolerance.

Subsequently, to determine the association between microorganisms enriched in saline–alkali soil and soybean stress physiological indicators and dry matter, we performed correlation analysis between the top 50 abundant genera in rhizosphere soil and representative physiological indicators (Figure 4e). In the results, *Bradyrhizobium* was negatively correlated with MDA, while *Streptomyces* was positively correlated with MDA. Genera such as *Nocardioides*, *Kribbella*, *Solirubrobacter*, *Conexibacter*, and *Phytoactinopolyspora* were positively correlated with one or more indicators besides MDA. *Streptomyces*, unclassified *Candidatus Eisenbacteria*, *Gemmatimonas*, *Gemmatirosa*, Candidatus *Gaiella silicea*, *Sphingomonas*, and *Roseisolibacter* were negatively correlated with one or more indicators besides MDA. Genera such as Candidatus *Gaiella silicea*, *Bradyrhizobium*, and *Solirubrobacter* were correlated with more than three indicators and may play important roles in the saline–alkali rhizosphere.

### 2.6. Rhizosphere Marker Microorganisms of Different Varieties Under Saline–Alkali Stress

To identify the differential rhizosphere microbial species between the two soybean varieties with different tolerances under saline–alkali conditions, we performed Linear Discriminant Analysis Effect Size (LEfSe) analysis on the abundance of rhizosphere microorganisms in TA and SA, using LDA > 2, *p* < 0.05 as screening criteria to find differential microorganisms between the two groups. The analysis revealed that the two groups had distinct phylogenetic compositions and identified multiple significantly representative biomarkers.

**Figure 5:**
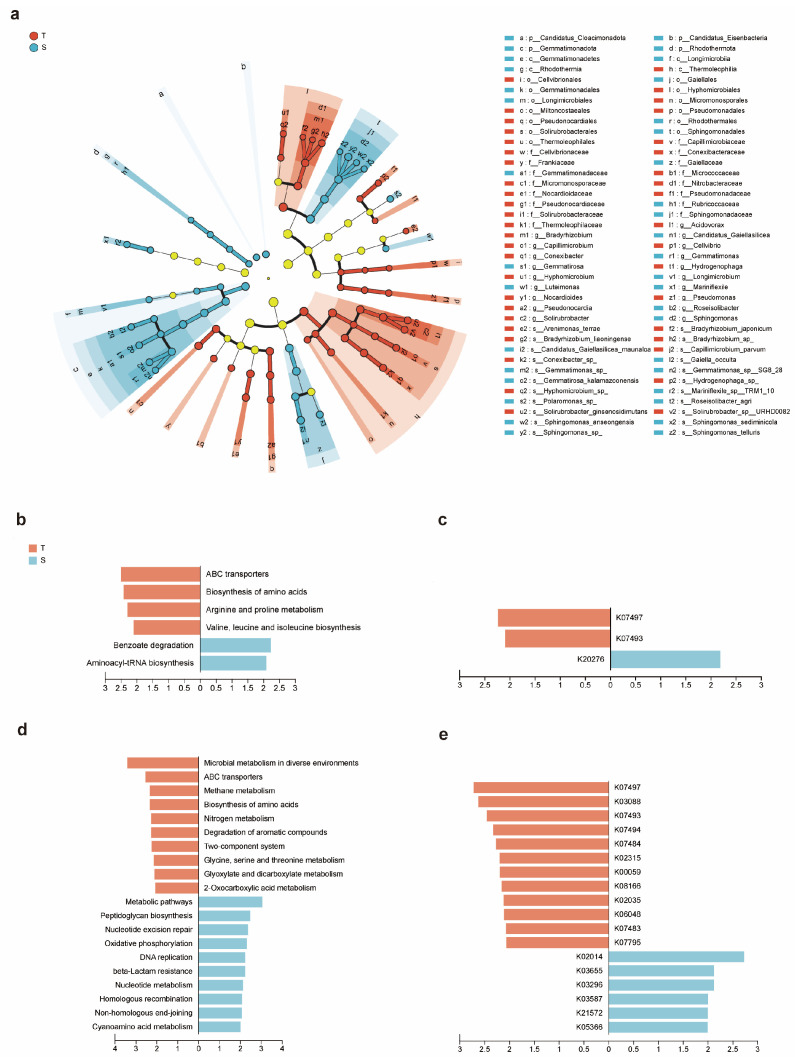
Rhizosphere soil marker microorganisms and functional differences in soybeans with different saline–alkali tolerances. (**a**) Hierarchical classification diagram of rhizosphere marker microorganisms for TA and SA, yellow circles indicate species with no significant difference. (**b**) Differences in the overall rhizosphere microbiome at KEGG database Level 3. (**c**) Differences in the overall rhizosphere microbiome at KEGG database KO level. (**d**) Differences in rhizosphere marker microorganisms at KEGG database Level 3. (**e**) Differences in rhizosphere marker microorganisms at KEGG database KO level. Note: TA represents the saline–alkali-tolerant soybean variety, SA represents the sensitive soybean variety. LEfSe analysis standard is LDA > 2, *p* < 0.05.

The biomarkers enriched in the TA group were predominantly led by multiple taxa from the phylum *Actinomycetota* (Figure 5a). At the class level, the class *Thermoleophilia* was the most significant marker taxon for the TA group (LDA = 3.06, *p* < 0.05). Further analysis showed that the order *Solirubrobacterales* under this class and its family *Solirubrobacteraceae* (including the genus *Solirubrobacter* and its species *S. ginsenosidimutans* and *S.* sp. URHD0082) were specifically enriched in the TA group. Additionally, nitrogen-fixing related taxa under the order *Hyphomicrobiales* (belonging to the class *Alphaproteobacteria*, phylum *Pseudomonadota*), such as the family *Nitrobacteraceae* and its genus *Bradyrhizobium* (including *B.* sp. and *B.liaoningense*), and the genus *Hyphomicrobium* of the family *Hyphomicrobiaceae*, were also key marker bacteria in the TA group. Notably, genera such as *Bradyrhizobium* and *Solirubrobacter*, which showed significant negative correlation with the stress damage indicator MDA, were precisely the taxa specifically enriched in the tolerant variety TA, suggesting their potential involvement in mitigating stress damage.

The marker microorganisms enriched in the SA group showed different enrichment characteristics from the TA microbial community. The order *Sphingomonadales* and its family *Sphingomonadaceae* (including the genus *Sphingomonas* and its multiple species such as *S*. sp., *S*. *anseongensis*, *S*. *sediminicola*) from the phylum *Pseudomonadota* were the most representative markers in the SA group (LDA > 3.5, *p* < 0.05). Simultaneously, the phylum *Gemmatimonadota* and its subordinate class *Gemmatimonadetes*, order *Gemmatimonadales* (including genera such as *Gemmatimonas*, *Gemmatirosa*, and *Roseisolibacter*) were also significantly enriched in the SA group. Furthermore, some known taxa common in barren or stressed environments, such as the phylum Candidatus *Cloacimonadota*, Candidatus *Eisenbacteria*, and the phylum *Rhodothermota*, were also specific marker bacteria for the SA group.

### 2.7. Functional Differences in Rhizosphere Biomarkers Between Varieties

To deeply explore the differences in rhizosphere microorganisms between TA and SA at the gene and functional levels, we functionally annotated the rhizosphere microorganisms of TA and SA using the KEGG database at Level 3 and KO levels, respectively, and performed LEfSe analysis on the overall rhizosphere microbiome and the rhizosphere marker microorganisms of TA and SA separately (using LDA > 2, *p* < 0.05 as the standard) to find KEGG functional pathways with significant abundance differences.

The rhizosphere microbial community of the TA group significantly enriched core functions related to amino acid metabolism and nutrient transport: Biosynthesis of amino acids, Valine, leucine and isoleucine biosynthesis, Arginine and proline metabolism, ABC transporters, while the microbial community of the SA group significantly enriched functions related to aromatic compound metabolism and basic protein synthesis processes: Benzoate degradation and Aminoacyl-tRNA biosynthesis (Figure 5b). At the KO level, the TA group enriched two KOs, K07497 and K07493, both putative transposases, while the SA group enriched one KO, K20276 (*bapA*, large repetitive protein). Currently, there are no literature reports linking these enriched KOs to saline–alkali resistance (Figure 5c). Integrating the above, overall, saline–alkali-tolerant soybeans showed more active amino acid metabolism, while sensitive soybeans had stronger functions in pathways like aromatic compound metabolism and protein synthesis, but the functional differences at the KO level were small.

The TA rhizosphere marker microorganisms mainly enriched 10 pathways at Level 3, including amino acid and nitrogen metabolism, core carbon metabolism and energy production, and signal sensing ABC transporters, while the SA group enriched 10 pathways at Level 3, including DNA repair, drug resistance, and basic metabolism (Figure 5d). The functions significantly enriched in the TA rhizosphere marker microorganisms at the KO level mainly included the following categories (Figure 5e): (1) transposase-related genes (transposase/putative transposase) (K07483, K07484, K07493, K07494, and K07497); (2) substance transport and metabolism related: *tctC* (K07795): membrane protein of the tricarboxylic acid transport system, may promote organic acid uptake and improve carbon source utilization. *ABC.PE.S* (K02035): Peptide/nickel transport system substrate-binding protein, can enhance nitrogen source and metal ion utilization. *mmr* (K08166): MFS efflux pump, drug resistance/harmful substance excretion. *gshA* (K06048): Glutamate-cysteine ligase, synthesizes the key antioxidant molecule glutathione. *fabG* (K00059): Fatty acid synthase (3-oxoacyl-ACP reductase), involved in membrane lipid metabolism. *dnaC* (K02315): DNA replication initiation protein. (3) Stress response and regulation: *rpoE* (K03088): involved in cell membrane/outer membrane stress response. The functions significantly enriched in the SA rhizosphere marker microorganisms mainly included the following categories: (1) cell wall/cell division related: *mrcA* (K05366): penicillin-binding protein 1A, involved in cell wall synthesis. *ftsI* (K03587): Penicillin-binding protein 3, involved in cell division. (2) DNA repair and recombination: *recG* (K03655): DNA helicase, involved in repair and recombination. (3) Substance transport: *TC.FEV.OM* (K02014): Iron complex outer membrane receptor protein. *susD* (K21572): Starch-binding outer membrane protein, involved in complex polysaccharide degradation. *TC.HAE1* (K03296): Hydrophobic/hydrophilic efflux pump (often in Gram-negative bacteria), often associated with drug/toxin efflux. In summary, the functional profile of the rhizosphere marker microbial community of the tolerant soybean leans towards genome plasticity (multiple transposase-related genes), stress resistance, oxidative stress defense, and enhanced substance transport capacity, facilitating rapid microbial adaptation to saline–alkali stress and providing stable metabolic support to the host. The functional profile of the rhizosphere marker microorganisms of the salt-sensitive soybean leans more towards basic growth (cell wall synthesis, division), nutrient competition (iron, starch), and DNA repair.

## 3. Discussion

Photosynthesis is a dynamic process that responds to fluctuations in sunlight, temperature, water availability, and CO_2_ on time scales ranging from seconds to minutes. Over longer time scales, changes in environmental cues (abiotic stress signals) induce adaptive responses, triggering signaling cascades from the nucleus to the cytoplasm and ultimately to the chloroplast, thereby altering the photosynthetic process [16]. Photosynthesis can be regarded as an indicator of plant physiological status; typically, the first physiological response to abiotic stress is a decline in photosynthetic activity. Chlorophyll fluorescence serves as a sensitive probe of photosynthetic performance, reflecting subtle changes in electron and energy transfer among electron carriers during the light reactions [17]. In this study, we first observed apparent yellowing of functional leaves and a marked decrease in chlorophyll content (Figure 1). Chlorophyll is the fundamental pigment of photosynthesis, responsible for light absorption, transfer, and conversion [18]. Chlorophyll synthesis is a high-priority metabolic process in plants; thus, the reduced chlorophyll content in the functional leaves of the salt-sensitive variety indicates severe disruption of its synthetic pathway [19]. In the fluorescence OJIP kinetics curve, the fluorescence intensities at the K, J, and I phases of the sensitive variety increased significantly, and both PI_ABS_ and φP_o_ (Fv/Fm) differed markedly from those of the tolerant variety. The overall performance of PSII was suppressed, the electron transport process was hindered, and the increase in W_k_ value further suggested potential structural damage to the oxygen-evolving complex (OEC) (Figure 2). Furthermore, by analyzing fluorescence parameters such as M_o_, S_m_, and φE_o_, it was found that, compared with the tolerant variety, the PSII acceptor-side electron pool capacity (S_m_) decreased in the functional leaves of the sensitive variety, the probability of electron transfer from QA^−^ to subsequent acceptors (φE_o_) decreased, and unutilized energy led to accelerated QA reduction (M_o_). Moreover, the reaction center activity (RC/CS_m_) of the sensitive variety also decreased, while energy dissipation per reaction center (DIo/RC) increased and electron transport rate per reaction center (ETo/RC) decreased significantly (Figure 2). These results are consistent with previous studies [20,21]. In summary, both the upstream OEC of the PSII electron transport chain and the plastoquinone (PQ) bridge connecting to the downstream Cyt b6/f complex were affected by stress, resulting in impaired electron transport throughout the light reactions; the overall decline in PSII function was more pronounced in the sensitive variety. The MDA content in plant roots reflects the extent of root cell membrane damage under saline–alkali stress [7]. Root damage in the tolerant variety under stress was significantly less severe than in the sensitive variety (Figure 3). Antioxidant enzyme activity analysis showed that the roots of the tolerant variety exhibited higher antioxidant enzyme activities and a stronger reactive oxygen species (ROS) scavenging capacity, thereby more effectively maintaining ROS homeostasis under saline–alkali stress [22]. These differences in physiological indicators indicate that the saline–alkali-tolerant variety effectively balanced ROS levels by maintaining high antioxidant system activity, thereby protecting the integrity of biomembrane structures [23]. Furthermore, its higher root vitality facilitates the absorption and transport of water and nutrients, providing greater resource availability to the aboveground parts, thereby maintaining photosynthetic capacity. The coordinated responses of aboveground tissues and roots to stress directly explain the greater dry matter accumulation in the saline–alkali-tolerant variety.

Saline–alkali soil differs markedly from conventional soil in its physicochemical properties (Table 1). In addition to its high pH, the concentrations of soluble salt ions such as Na^+^, Ca^2+^, and HCO_3_^−^ in saline–alkali soil are much higher than those in conventional soil—approximately 4.9-, 2.6-, and 2.7-fold higher, respectively. This harsh environment exerts strong natural selection pressure on the soil microbial community, profoundly influencing the structure of the rhizosphere microbiome [24]. In this study, no significant difference in alpha diversity (based on the ACE index) was detected between rhizosphere microbial communities in saline–alkali and conventional soils. However, beta diversity analysis (PCoA based on Bray–Curtis distance) revealed that PC1 primarily reflected the impact of saline–alkali stress on rhizosphere microbial community structure (Figure 4). PC2 further separated the rhizosphere microbial communities by soybean genotype. Under normal soil conditions, the rhizosphere microbial communities of the tolerant (T) and sensitive (S) varieties were clearly distinct, indicating that different soybean genotypes possess the ability to selectively recruit microorganisms. In contrast, under saline–alkali stress, this genotype-specific recruitment capacity was weakened, leading to a homogenization of the rhizosphere microbial community structures between the two varieties [11]. Although alpha diversity did not differ significantly between rhizosphere soils from saline–alkali and normal conditions, substantial differences in species abundance were observed. At the genus level, salt-tolerant taxa such as *Rubrobacter* and *Streptomyces* were significantly enriched in saline–alkali rhizosphere soils. Conversely, genera commonly dominant in conventional soil with beneficial ecological functions—such as *Sphingomonas*, which degrades recalcitrant pollutants, and *Bradyrhizobium*, which fixes nitrogen and promotes soybean growth—showed significantly reduced abundances under saline–alkali stress (Figure 4). These results are consistent with previous studies [25,26,27]. Correlation analysis between rhizosphere microbial genera, physiological indicators, and dry matter accumulation in saline–alkali soil revealed that *Bradyrhizobium*, *Nocardioides*, *Kribbella*, *Solirubrobacter*, and *Conexibacter* were positively correlated with one or more physiological traits, suggesting beneficial effects. This finding aligns with previous reports showing that *Bradyrhizobium* enhances the antioxidant capacity of leguminous crops [28]. *Nocardioides*, an actinobacterial genus, plays a key role in rhizosphere soil due to its remarkable ability to degrade recalcitrant pollutants—including aromatic compounds, hydrocarbons, halogenated alkanes, nitrogen heterocycles, and polyester polymers—at rates higher than most other bacterial strains [29]. *Kribbella* has been reported to increase soil potassium and phosphorus availability under saline–alkali conditions and to promote plant growth [30]. *Solirubrobacter* is widely distributed in plant rhizospheres, although its ecological functions remain poorly characterized, suggesting that it may play an important yet underexplored role in saline–alkali environments. Overall, the correlation analysis underscores the strong coherence between rhizosphere microbial composition and soybean physiological responses, indicating a close coordination between plant performance and its associated microbiome under saline–alkali stress.

Although saline–alkali stress caused an overall convergence of rhizosphere microbial community structures, differential enrichment analysis still identified significantly distinct microbial taxa between the two soybean varieties. At multiple taxonomic levels, both the tolerant (T) and sensitive (S) varieties enriched different microbial species, suggesting that they respond to saline–alkali stress by selectively recruiting distinct microorganisms (Figure 5). Among the taxa significantly enriched in the T variety, *Bradyrhizobium* sp. and *Bradyrhizobium liaoningense* were particularly notable. These are symbiotic nitrogen-fixing bacteria that serve as bio-nitrogen fertilizers (BNFs), supplying nitrogen to soybean plants and enhancing root antioxidant capacity [26]. Considering the critical role of nitrogen in chlorophyll biosynthesis, and in light of the correlation analysis results, we speculate that the higher SPAD value (Figure 1b) and superior photosynthetic efficiency observed in the tolerant variety at the seedling stage may be associated with its ability to recruit a greater abundance of nitrogen-fixing bacteria [31]. In addition, several other bacterial taxa enriched in the T variety—such as *Hydrogenophaga* sp., *Aquabacterium pictum*, *Solirubrobacter ginsenosidimutans*, and *Solirubrobacter* sp. URHD0082—may contribute to the stability of the rhizosphere microenvironment and improve nutrient availability by degrading organic matter and pollutants. Functional enrichment analysis of marker microorganisms further revealed that the T rhizosphere was enriched in genes associated with transposases, oxidative stress responses (e.g., glutamate–cysteine ligase *gshA*, K06048), stress response regulation (sigma factor *rpoE*, K03088), and resistance to antibiotics and heavy metals (e.g., multidrug resistance protein *mmr*, K08166; peptide/nickel transport protein *ABC.PE.S*, K02035) [32,33,34]. In contrast, the S group was enriched in genes primarily related to cell wall synthesis and division (e.g., *mrcA*, K05366; *ftsI*, K03587), iron acquisition (e.g., iron complex outer membrane receptor protein, K02014), DNA repair and recombination (e.g., *recG*, K03655), and carbon source utilization and transport (e.g., *susD*, K21572; *TC.HAE1*, K03296). These differences suggest that the marker microorganisms of T and S adopted distinct adaptive strategies under saline–alkali stress: microorganisms associated with T tended toward active stress resistance and environmental detoxification, whereas those associated with S focused more on maintaining basal metabolic functions and nutrient uptake. Despite these genotype-specific differences, the overall functional enrichment analysis indicated that the T and S rhizosphere microbiomes were functionally similar, consistent with the PCoA results. This suggests that under saline–alkali stress, the rhizosphere microbiomes of different soybean genotypes may undergo functional convergent evolution, and the plant–microbe interaction tends toward functional redundancy rather than dependence on a few key microbial taxa [35].

**Figure 6:**
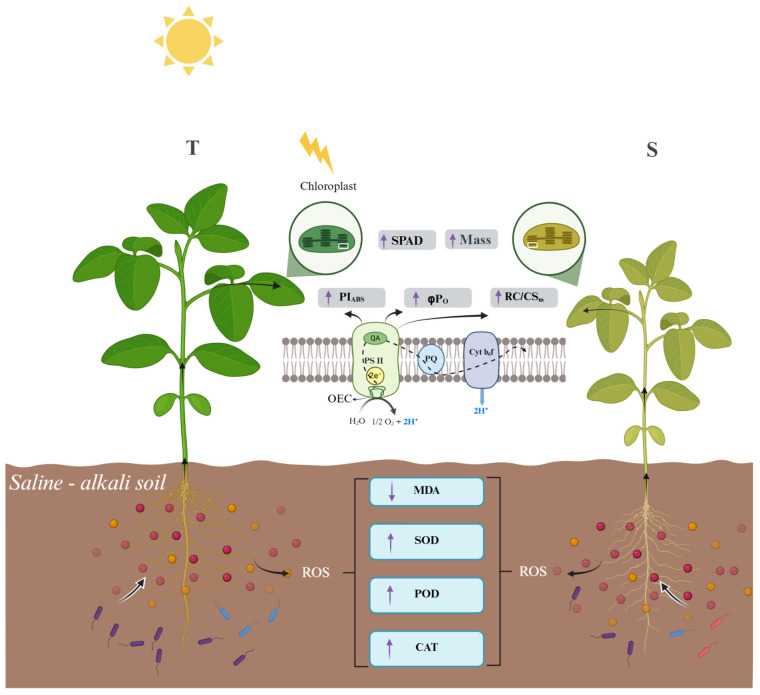
Schematic diagram of the interaction between physiology and rhizosphere microbiome in soybeans with different saline–alkali tolerances. Arrows in the figure indicate the direction of change in physiological indicators for the tolerant variety compared to the sensitive variety, T for tolerant variety and S for sensitive variety.

Under saline–alkali stress, soybean roots experience ion toxicity and osmotic imbalance, leading to the accumulation of reactive oxygen species (ROS), as indicated by increased malondialdehyde (MDA) levels. The tolerant variety activates stronger antioxidant defenses, maintaining root activity and membrane stability. Sustained root function ensures the continuous transport of water and nutrients to the shoots, stabilizing chlorophyll content and protecting the photosynthetic apparatus. Efficient photosynthesis supplies assimilates to the roots, supporting metabolism and enhancing the secretion of organic compounds into the rhizosphere. These exudates promote the enrichment of beneficial microorganisms that, in turn, improve root performance and stress adaptation. Together, these reciprocal interactions form a coordinated system linking root physiology, the rhizosphere microbiome, and photosynthetic function, enabling soybean plants to better tolerate saline–alkali stress (Figure 6).

## 4. Materials and Methods

### 4.1. Experimental Design

Experimental soils were collected from saline–alkali land and conventional farmland in Baicheng City, Jilin Province, China (45°28′ N, 123°12′ E). The sampling depth was the topsoil layer (0–20 cm). After air-drying, crushing, and sieving, 3 kg of soil was accurately weighed and placed into each pot. Prior to the formal experiment, all soils underwent a two-week equilibration and stabilization period, after which their initial physicochemical properties were determined (Table 1). Two soybean (*Glycine max* [L.] *Merr.*) varieties with contrasting saline–alkali tolerances were used: the tolerant variety ‘Jiyu 99’ (T) and the sensitive variety ‘Jiyu 83’ (S). These varieties were selected by the Soybean Research Institute of the Jilin Academy of Agricultural Sciences based on three years of field screening in saline–alkali land in Baicheng City. Soybeans were sown in pots containing either saline–alkali soil (A) or non-saline soil (N). The experiment consisted of four treatments: (1) tolerant variety + saline–alkali soil (TA), (2) tolerant variety + non-saline soil (TN), (3) sensitive variety + saline–alkali soil (SA), and (4) sensitive variety + non-saline soil (SN). All pots were cultivated in a controlled greenhouse environment with a temperature of 25 °C, relative humidity of 60–70%, and a 12 h light/12 h dark photoperiod. Watering was performed once every two days after sowing, and once per day after the emergence of true leaves. Approximately 200 mL of water was applied per pot each time, with slight adjustments according to actual soil moisture, but the amount per pot was kept consistent each time.

Soil physicochemical properties were determined as follows: Electrical conductivity (EC) was measured using a DDS-307A conductivity meter (INESA Scientific Instrument Ltd., Shanghai, China) by mixing 5 g of soil with 25 mL of deionized water. Soil pH was measured using a PHS-2F pH meter (INESA Scientific Instrument Ltd., Shanghai, China) after mixing 10 g of soil with 25 mL of deionized water. Both soil suspensions were stirred thoroughly, allowed to stand for 30 min, and the supernatants were subsequently used for measurement. Water-soluble ions (Na^+^, K^+^, Ca^2+^, Mg^2+^, Cl^−^, HCO_3_^−^, CO_3_^2−^, and SO_4_^2−^), available nitrogen (AN), available phosphorus (AP), available potassium (AK), and soil organic matter (SOM) were determined following standard protocols described in Methods of Soil Analysis, Part 3: Chemical Methods [36].

### 4.2. Determination of Physiological Parameters and Dry Matter

#### 4.2.1. Functional Leaf Chlorophyll Content and OJIP Fluorescence Measurement

At the V3 stage (approximately 32 days after sowing), the SPAD value of functional leaves was measured using a SPAD-502 chlorophyll meter (Konica Minolta, Tokyo, Japan). The OJIP fast fluorescence induction kinetics curve was recorded using a portable plant efficiency analyzer (Pocket-PEA, Hansatech Instruments Ltd., King’s Lynn, UK).

Before measurement, selected leaves were dark-adapted for 30 min using the instrument’s dedicated leaf clips. The OJIP fluorescence transient was induced by a red light pulse (wavelength 650 nm, light intensity 3500 μmol·m^−2^·s^−1^) lasting 1 s. In the OJIP curve, O represents the initial fluorescence; K (~300 μs), J (~2–3 ms), and I (~30 ms) are intermediate characteristic points; and P (500 ms–1 s) represents the fluorescence peak [37]. Fluorescence data were analyzed using the JIP-test method to derive biophysical parameters reflecting the structure and function of photosystem II (PSII) [38]. The original fluorescence parameters included Fo (initial fluorescence), F_k_ (fluorescence at the K point, 300 μs), F_J_ (fluorescence at the J point, 2 ms), F_I_ (fluorescence at the I point, 30 ms), Fm (maximum fluorescence when all reaction centers are closed), and Ft (fluorescence intensity at time *t*). The relative variable fluorescence intensity at time *t* was calculated as: Vt = (Ft − Fo)/(Fm − Fo). Based on the above parameters, the following indicators were further calculated: W_k_ = (F_k_ − Fo)/(F_J_ − Fo), maximum photochemical quantum efficiency of PSII φPo = Fv/Fm = (Fm − Fo)/Fm, initial slope of fluorescence rise M_o_ = 4 · (F300μs − Fo)/(Fm − Fo), plastoquinone (PQ) pool size per reaction center S_m_ = Area/(Fm − Fo), number of active reaction centers per unit cross-sectional area RC/CS = φP_o_ · (ABS/CS), probability of electron transport beyond QA^−^ ψo = 1 − V_J_, comprehensive performance index reflecting PSII photochemical performance PI_ABS_ = [RC/ABS] · [φPo/(1 − φPo)] · [ψo/(1 − ψo)].

#### 4.2.2. Root Malondialdehyde Content and Antioxidant Enzyme Activity Determination

Kits produced by Beijing Solarbio Science and Technology Co., Ltd. (Beijing, China) were used to determine root malondialdehyde (MDA) content and the activities of superoxide dismutase (SOD), peroxidase (POD), and catalase (CAT), strictly following the kit instructions. MDA content was determined using the thiobarbituric acid (TBA) method (Cat. No. BC0020), reading absorbance at 532 nm and 600 nm. SOD activity was determined by the nitroblue tetrazolium (NBT) photoreduction method (Cat. No. BC0170), calculating enzyme activity based on the inhibition rate of photoreduction at 560 nm. POD activity was determined using the guaiacol method (Cat. No. BC0090), monitoring the increase in absorbance at 470 nm due to tetraguaiacol formation. CAT activity was determined by the ultraviolet absorption method (Cat. No. BC0200), monitoring the decrease in absorbance at 240 nm due to H_2_O_2_ decomposition. All absorbance measurements were completed using a UH5300 model spectrophotometer manufactured by Hitachi High-Technologies Corporation (Minato-ku, Tokyo, Japan). Enzyme activity was expressed in units per gram fresh weight per minute (U/g FW/min). Specific operations are detailed in the respective product manuals.

#### 4.2.3. Plant Dry Matter Determination

After completing the physiological indicator measurements for leaves and roots, the entire plant was placed in an 80 °C oven and dried to constant weight. The plant’s dry weight was then weighed and recorded.

### 4.3. Soil Sampling and Metagenomic Sequencing

#### 4.3.1. Rhizosphere Soil Collection

The complete soil block containing the root system was carefully removed from the pot. The soil block was gently broken, and the rhizosphere soil was loosened and detached by slight shaking. Subsequently, soil still tightly adhering to the root surface was carefully brushed off using a sterile soft brush and collected into cryotubes. Collected samples were immediately flash-frozen in liquid nitrogen and then transferred to a −80 °C ultra-low temperature freezer for long-term storage.

#### 4.3.2. DNA Extraction and Metagenomic Sequencing

Total genomic DNA was extracted from 0.5 g soil samples using the E.Z.N.A.^®^ Soil DNA Kit (Omega Bio-tek, Norcross, GA, USA) according to the manufacturer’s instructions. DNA concentration and purity were detected using a Synergy HTX microplate reader (Agilent Technologies, Inc., Santa Clara, CA, USA) and NanoDrop 2000 spectrophotometer (Thermo Fisher Scientific, Inc., Wilmington, DE, USA), respectively, and DNA integrity was assessed by 1% agarose gel electrophoresis. DNA was randomly fragmented to an average size of approximately 350 bp using a Covaris M220 Focused-ultrasonicator (Covaris LLC, Woburn, MA, USA) and paired-end sequencing libraries were constructed using the NEXTFLEX Rapid DNA-Seq Library Prep Kit (Bioo Scientific, Austin, TX, USA). Subsequent sequencing was performed at Majorbio Bio-Pharm Technology Co., Ltd. (Shanghai, China) using the Illumina NovaSeq™ X Plus platform (Illumina Inc., San Diego, CA, USA) and NovaSeq X Series 25B Reagent Kit (Illumina Inc., San Diego, CA, USA), strictly following the official Illumina operating procedures: https://www.illumina.com (accessed on 25 February 2025) for paired-end sequencing.

#### 4.3.3. Metagenomic Sequencing Data Processing

Data was analyzed on the free online platform of the Majorbio Cloud Platform https://www.majorbio.com (accessed on 20 September 2025) [38]. Briefly, fastp [39] (https://github.com/OpenGene/fastp, accessed on 25 February 2025, version 0.23.0) was used to trim adapters from raw sequencing reads and remove low-quality reads (length < 50 bp or average quality value < 20). Quality-filtered data were assembled using MEGAHIT [40] (https://github.com/voutcn/megahit, accessed on 25 February 2025, version 1.1.2). Contigs with length ≥ 300 bp were selected as the final assembly result. Open reading frames (ORFs) in each assembled contig were predicted using Prodigal [41] (https://github.com/hyattpd/Prodigal, accessed on 25 February 2025, version 2.6.3), and ORFs with length ≥ 100 bp were retrieved. A non-redundant gene catalog was constructed using CD-HIT [42] (http://weizhongli-lab.org/cd-hit/, accessed on 25 February 2025, version 4.6.1) with 90% sequence identity and 90% coverage. Gene abundance for specific samples was estimated using SOAPaligner [43] (https://github.com/ShujiaHuang/SOAPaligner, accessed on 25 February 2025, version soap2.21release) at 95% identity.

#### 4.3.4. Bioinformatics and Statistical Data Analysis

Non-redundant genes were aligned against the NCBI NR database using DIAMOND [44] (http://ab.inf.uni-tuebingen.de/software/diamond/, accessed on 25 February 2025, version 2.0.13) with an e-value cutoff of 1e-5 to obtain their best hit taxonomy. Similarly, functional annotation (KEGG) of non-redundant genes was obtained. Based on taxonomic and functional annotations and the abundance of non-redundant genes, differential analysis was performed at each taxonomic, functional, or gene level using the Kruskal–Wallis test. Physiological indicators and dry matter were subjected to *t*-tests under the same soil conditions, with different significance levels indicated by different numbers of asterisks: * represents *p* < 0.05, ** represents *p* < 0.01, *** represents *p* < 0.001, **** represents *p* < 0.0001.

## 5. Conclusions

This study investigated differences in the physiological characteristics of functional leaves and roots, as well as the rhizosphere microbial composition and function, between soybean varieties with contrasting salt tolerances. At the physiological level, the salt-tolerant variety exhibited higher chlorophyll content in functional leaves, superior photosynthetic performance, and stronger root antioxidant enzyme activity than the sensitive variety, demonstrating its pronounced saline–alkali tolerance and resulting in greater biomass accumulation.

In terms of the rhizosphere microbiome, soybean varieties with different saline–alkali tolerances harbored distinct marker microorganisms. Functional analyses of these marker taxa revealed certain differences in their adaptive responses to saline–alkali stress; however, the overall functional profiles of the rhizosphere microbiomes tended to converge, indicating that the contribution of individual marker species to enhanced soybean tolerance was relatively limited.

Integrating the physiological and microbial findings, this study suggests that within the soybean–microbe interaction system, the plant’s intrinsic physiological traits play a predominant role. Enhanced root vitality in the tolerant variety ensures the efficient transport of water and nutrients to aboveground tissues, thereby supporting photosynthetic activity in functional leaves. In turn, robust photosynthesis provides the metabolic foundation for root exudation, facilitating the recruitment of beneficial microorganisms. This coordinated interaction between soybean physiology and the rhizosphere microbiome underlies the superior saline–alkali resistance observed in the tolerant genotype.

## Figures and Tables

**Table 1:** Physicochemical properties of the experimental soil.

Soil	pH	Total Water-Soluble Salts (g/kg)	Electrical Conductivity (μS/cm)	Organic Matter (g/kg)	Available Potassium (mg/kg)
N	7.39	0.04	482.3	30.5	96
A	8.29	1.10	1217.7	24.78	144.39
Soil	Available nitrogen (mg/kg)	Available phosphorus (mg/kg)	Sodium ion (mg/kg)	Potassium ion (mg/kg)	Calcium ion (mg/kg)
N	129.76	55.74	22.32	4.21	49
A	145.18	13.04	109.45	12.28	129.53
Soil	Magnesium ion (mg/kg)	Sulfate (g/kg)	Carbonate (g/kg)	Bicarbonate (g/kg)	Chloride ion (g/kg)
N	14	<0.02	<0.01	0.19	0.02
A	17.97	0.02	0.01	0.51	0.03

Note: N is for normal soil, and A is for saline–alkali soil.

## Data Availability

The metagenomic sequencing data generated in this study has been deposited in the NCBI Sequence Read Archive database under accession number PRJNA1312967.

## Abbreviations

The following abbreviations are used in this manuscript:

PSII	Photosystem II
ROS	Reactive Oxygen Species
MDA	Malondialdehyde
SOD	Superoxide Dismutase
POD	Peroxidase
CAT	Catalase
SPAD	Chlorophyll content
Fo	Minimal fluorescence (all reaction centers open)
F_k_	Fluorescence intensity at the K-step
F_J_	Fluorescence intensity at the J-step
F_I_	Fluorescence intensity at the I-step
Fm	Maximal fluorescence (all reaction centers closed)
Ft	Fluorescence at time t
Vt	Relative variable fluorescence at time t
W_k_	Damage index to the Oxygen-Evolving Complex
Fv/Fm	Maximum quantum efficiency of PSII photochemistry
φP_o_	Maximum quantum yield of primary photochemistry
PI_ABS_	Performance index on absorption basis
φE_o_	Quantum yield of electron transport beyond QA^−^
M_o_	Initial slope of the fluorescence rise
S_m_	Normalized area above the OJIP curve (proportional to the plastoquinone pool size)
RC/CS_m_	Reaction centers per cross-section
DIo/RC	Dissipated energy flux per reaction center
ETo/RC	Electron transport flux beyond QA^−^ per reaction center
OEC	Oxygen-evolving complex
QA	Primary quinone electron acceptor of PSII
PQ	Plastoquinone

## References

[1] Corwin D.L. (2021). Climate Change Impacts on Soil Salinity in Agricultural Areas. Eur. J. Soil Sci..

[2] Singh A. (2022). Soil Salinity: A Global Threat to Sustainable Development. Soil Use Manag..

[3] zaaboul F., Zhao Q., Xu Y., Liu Y. (2022). Soybean Oil Bodies: A Review on Composition, Properties, Food Applications, and Future Research Aspects. Food Hydrocoll..

[4] van Zelm E., Zhang Y., Testerink C. (2020). Salt Tolerance Mechanisms of Plants. Annu. Rev. Plant Biol..

[5] Athar H.-u.-R., Zafar Z.U., Ashraf M. (2015). Glycinebetaine Improved Photosynthesis in Canola under Salt Stress: Evaluation of Chlorophyll Fluorescence Parameters as Potential Indicators. J. Agron. Crop Sci..

[6] Soliman W.S., Fujimori M., Tase K., Sugiyama S. (2011). Oxidative Stress and Physiological Damage under Prolonged Heat Stress in C3 Grass Lolium Perenne: Oxidative Stress and Heat Stress in C3 Grass. Grassl. Sci..

[7] Gaweł S., Wardas M., Niedworok E., Wardas P. (2004). Malondialdehyde (MDA) as a lipid peroxidation marker. Wiad Lek.

[8] Dumanović J., Nepovimova E., Natić M., Kuča K., Jaćević V. (2021). The Significance of Reactive Oxygen Species and Antioxidant Defense System in Plants: A Concise Overview. Front. Plant Sci..

[9] Aung T.T., Shi F., Zhai Y., Xue J., Wang S., Ren X., Zhang X. (2022). Acidic and Alkaline Conditions Affect the Growth of Tree Peony Plants via Altering Photosynthetic Characteristics, Limiting Nutrient Assimilation, and Impairing ROS Balance. Int. J. Mol. Sci..

[10] Geilfus C.-M. (2018). Review on the Significance of Chlorine for Crop Yield and Quality. Plant Sci..

[11] Sasse J., Martinoia E., Northen T. (2018). Feed Your Friends: Do Plant Exudates Shape the Root Microbiome?. Trends Plant Sci..

[12] Gil T., Teixeira R., Sousa A., d’Oliveira Palmeiro M.A., Cruz Coimbra de Matos A., Niza Costa M., Ferrer M.V., Rodrígues dos Santos A.S., Sequero López C., Rebelo Romão I. (2023). Isolation and Characterization of Culturable Osmotolerant Microbiota in Hypersaline and Hypergypsic Soils as New Treatment for Osmotic Stress in Plants. Soil Syst..

[13] Kumar A., Singh S., Gaurav A.K., Srivastava S., Verma J.P. (2020). Plant Growth-Promoting Bacteria: Biological Tools for the Mitigation of Salinity Stress in Plants. Front. Microbiol..

[14] Zhang J., Xu T., Liu Y., Chen T., Zhang Q., Li W., Zhou H., Zhang Y., Zhang Z. (2022). Molecular Insights into Salinity Responsiveness in Contrasting Genotypes of Rice at the Seedling Stage. Int. J. Mol. Sci..

[15] Bont Z., Züst T., Arce C.C.M., Huber M., Erb M. (2020). Heritable Variation in Root Secondary Metabolites Is Associated with Recent Climate. J. Ecol..

[16] Eberhard S., Finazzi G., Wollman F.-A. (2008). The Dynamics of Photosynthesis. Annu. Rev. Genet..

[17] Baker N.R. (2008). Chlorophyll Fluorescence: A Probe of Photosynthesis In Vivo. Annu. Rev. Plant Biol..

[18] Wei Z., Duan F., Sun X., Song X., Zhou W. (2021). Leaf Photosynthetic and Anatomical Insights into Mechanisms of Acclimation in Rice in Response to Long-term Fluctuating Light. Plant Cell Environ..

[19] Tanaka A., Tanaka R. (2006). Chlorophyll Metabolism. Curr. Opin. Plant Biol..

[20] Mehta P., Jajoo A., Mathur S., Bharti S. (2010). Chlorophyll *a* Fluorescence Study Revealing Effects of High Salt Stress on Photosystem II in Wheat Leaves. Plant Physiol. Biochem..

[21] Zushi K., Matsuzoe N. (2017). Using of Chlorophyll a Fluorescence OJIP Transients for Sensing Salt Stress in the Leaves and Fruits of Tomato. Sci. Hortic..

[22] Gomes B.R., Siqueira-Soares R.D.C., Santos W.D.D., Marchiosi R., Soares A.R., Ferrarese-Filho O. (2014). The Effects of Dopamine on Antioxidant Enzymes Activities and Reactive Oxygen Species Levels in Soybean Roots. Plant Signal. Behav..

[23] Zhang H., Yu F., Xie P., Sun S., Qiao X., Tang S., Chen C., Yang S., Mei C., Yang D. (2023). A Gγ Protein Regulates Alkaline Sensitivity in Crops. Science.

[24] Yang D., Tang L., Cui Y., Chen J., Liu L., Guo C. (2022). Saline-Alkali Stress Reduces Soil Bacterial Community Diversity and Soil Enzyme Activities. Ecotoxicology.

[25] Chen M.-Y., Wu S.-H., Lin G.-H., Lu C.-P., Lin Y.-T., Chang W.-C., Tsay S.-S. (2004). *Rubrobacter taiwanensis* sp. Nov., a Novel Thermophilic, Radiation-Resistant Species Isolated from Hot Springs. Int. J. Syst. Evol. Microbiol..

[26] Saranraj P., Sivasakthivelan P., Al-Tawaha A.R.M., Sudha A., Al-Tawaha A.R., Sirajuddin S.N. (2021). Hastang Diversity and Evolution of Bradyrhizobium Communities Relating to Soybean Cultivation: A Review. IOP Conf. Ser. Earth Environ. Sci..

[27] White D.C., Sutton S.D., Ringelberg D.B. (1996). The Genus Sphingomonas Physiology and Ecology. Curr. Opin. Biotechnol..

[28] Rodrigues A.C., Bonifacio A., Antunes J.E.L., da Silveira J.A.G., Figueiredo M.d.V.B. (2013). Minimization of Oxidative Stress in Cowpea Nodules by the Interrelationship between *Bradyrhizobium* sp. and Plant Growth-Promoting Bacteria. Appl. Soil Ecol..

[29] Ma Y., Wang J., Liu Y., Wang X., Zhang B., Zhang W., Chen T., Liu G., Xue L., Cui X. (2023). Nocardioides: “Specialists” for Hard-to-Degrade Pollutants in the Environment. Molecules.

[30] Yang L., Yang K. (2020). Biological Function of *Klebsiella variicola* and Its Effect on the Rhizosphere Soil of Maize Seedlings. PeerJ.

[31] Ma B.L., Morrison M.J., Voldeng H.D. (1995). Leaf Greenness and Photosynthetic Rates in Soybean. Crop Sci..

[32] Khedr A.H.A. (2003). Proline Induces the Expression of Salt-Stress-Responsive Proteins and May Improve the Adaptation of *Pancratium maritimum* L. to Salt-Stress. J. Exp. Bot..

[33] Poustini K., Siosemardeh A., Ranjbar M. (2007). Proline Accumulation as a Response to Salt Stress in 30 Wheat (*Triticum aestivum* L.) Cultivars Differing in Salt Tolerance. Genet. Resour. Crop Evol..

[34] Ding W., Zhang W., Alikunhi N.M., Batang Z., Pei B., Wang R., Chen L., Al-Suwailem A., Qian P.-Y. (2019). Metagenomic Analysis of Zinc Surface–Associated Marine Biofilms. Microb. Ecol..

[35] Li H., La S., Zhang X., Gao L., Tian Y. (2021). Salt-Induced Recruitment of Specific Root-Associated Bacterial Consortium Capable of Enhancing Plant Adaptability to Salt Stress. ISME J..

[36] Sparks D.L., Page A.L., Helmke P.A., Loeppert R.H. (2020). Methods of Soil Analysis, Part 3: Chemical Methods.

[37] Chen S., Yang J., Zhang M., Strasser R.J., Qiang S. (2016). Classification and Characteristics of Heat Tolerance in *Ageratina adenophora* Populations Using Fast Chlorophyll a Fluorescence Rise O-J-I-P. Environ. Exp. Bot..

[38] Han C., Shi C., Liu L., Han J., Yang Q., Wang Y., Li X., Fu W., Gao H., Huang H. (2024). Majorbio Cloud 2024: Update Single-cell and Multiomics Workflows. iMeta.

[39] Chen S., Zhou Y., Chen Y., Gu J. (2018). Fastp: An Ultra-Fast All-in-One FASTQ Preprocessor. Bioinformatics.

[40] Li D., Liu C.-M., Luo R., Sadakane K., Lam T.-W. (2015). MEGAHIT: An Ultra-Fast Single-Node Solution for Large and Complex Metagenomics Assembly via Succinct *de Bruijn* Graph. Bioinformatics.

[41] Hyatt D., Chen G.-L., LoCascio P.F., Land M.L., Larimer F.W., Hauser L.J. (2010). Prodigal: Prokaryotic Gene Recognition and Translation Initiation Site Identification. BMC Bioinform..

[42] Fu L., Niu B., Zhu Z., Wu S., Li W. (2012). CD-HIT: Accelerated for Clustering the next-Generation Sequencing Data. Bioinformatics.

[43] Li R., Li Y., Kristiansen K., Wang J. (2008). SOAP: Short Oligonucleotide Alignment Program. Bioinformatics.

[44] Buchfink B., Xie C., Huson D.H. (2015). Fast and Sensitive Protein Alignment Using DIAMOND. Nat. Methods.

